# New-Onset Diabetes Mellitus (NODM) After Liver Transplantation (LT): The Ultimate Non-diabetogenic Immunosuppressive Therapy

**DOI:** 10.7759/cureus.23635

**Published:** 2022-03-29

**Authors:** Ali R Chaitou, Surbhi Valmiki, Mrinaal Valmiki, Maria Zahid, Mohamed A Aid, Peter Fawzy, Safeera Khan

**Affiliations:** 1 Internal Medicine, California Institute of Behavioral Neurosciences & Psychology, Fairfield, USA; 2 Obstetrics and Gynecology, California Institute of Behavioral Neurosciences & Psychology, Fairfield, USA; 3 Psychiatry, California Institute of Behavioral Neurosciences & Psychology , Fairfield , USA; 4 Intensive Care Unit, California Institute of Behavioral Neurosciences & Psychology, Fairfield, USA; 5 Neurological Surgery, California Institute of Behavioral Neurosciences & Psychology, Fairfield, USA

**Keywords:** diabetes mellitus type 2, liver transplantation, nodm, nodat, nodalt, tacrolimus, cyclosporine-a, corticosteroids, immunosuppression

## Abstract

New-onset diabetes mellitus (NODM) is a common long-term complication after liver transplantation (LT). It is thought to be drug-induced in most cases, no matter the underlying disease that cause liver failure and indicated transplantation. Standard post-transplantation (PT) immunosuppressive regimens include prolonged use of calcineurin inhibitors (CNIs), namely tacrolimus (TAC), alongside corticosteroids to avoid acute and chronic graft rejection. This combination is well known for its diabetogenicity. Significant differences between the applied regimens stand out concerning the duration and dosages to prevent the metabolic side effects of these drugs in the long run without compromising the graft's survival. Studies were collected after an extensive research of PubMed database for this very specific topic using the following MeSH keywords in multiple combinations: "Liver Transplantation," "Diabetes Mellitus," "NODM," "Tacrolimus," "Cyclosporine A," and "Steroids." In addition, we used the same keywords for regular searches in Google Scholar. Only the relevant English human studies between 2010 and 2020 were collected except for review articles. Duplicates were eliminated using Mendeley software. Twelve relevant studies directly related to the targeted topic were collected and discussed, including five retrospective cohorts, four prospective cohorts, one clinical trial, one prospective pilot, and one case report. Their topics included primarily the factors increasing the risk of new-onset diabetes mellitus after liver transplantation (NODALT), TAC-based immunosuppression and its relative blood levels affecting the possible development of NODALT, the role of cyclosporine in substituting TAC regimen, and the effect of different steroids-avoiding protocols on the prevention of NODALT. The reviewed studies suggested that lowering the serum concentration of tacrolimus (cTAC) throughout the PT period and eliminating the corticosteroids regimen as early as possible, among other measures, can significantly impact the rate of emergence of NODM. This traditional review tackles the most recent studies about NODALT to establish a comprehensive view on this issue and guide clinicians and researchers for the safest immunosuppressive regimen to date, while maintaining a balanced metabolic profile.

## Introduction and background

"Without the organ donor, there is no story, no hope, no transplant. But when there is an organ donor, life springs from death, sorrow turns to hope, and a terrible loss becomes a gift", quotes a solid organ recipient for the united network for organ sharing (UNOS) [1]. Solid-organ transplantation sparkled in the sky of medicine in the twentieth century as a lifesaving surgery in patients suffering from refractory organ failure, but what always mattered most was the ability to overcome the challenges of keeping the grafted gift alive post-operatively, hence improving the recipient's quality of life and raising his newly born hope.

Liver transplantation (LT) is one of the major solid organ transplantations frequently conducted since pioneered in 1963 by Starzl, indicated majorly for hepatocellular carcinoma, hepatitis C infection, and alcoholic liver cirrhosis [2]. Impeded by different metabolic and non-metabolic complications, new-onset diabetes mellitus after liver transplantation (NODALT) is one of the common metabolic ones following LT that threatens the longevity of the graft and the recipient altogether, likely by increasing the risk of infection, cardiovascular disease, and graft rejection [3-5]. New-onset diabetes after transplant (NODAT) is defined by the American Diabetes Association (ADA) as new-onset diabetes in transplant recipients following organ transplantation, excluding patients with pre-transplant diabetes that was undiagnosed as well as post-transplantation (PT) hyperglycemia that resolves by the time of discharge [6]. Its incidence ranges from 17% to over 60% according to various studies [4-11]. Multiple factors are thought to take part in triggering its occurrences such as recipient's family history, high BMI, male sex, advanced age, tacrolimus (TAC)-based immunosuppression, corticosteroids use, hepatitis C infection, some different genetic and epigenetic variants, in addition to open donor hepatectomy in living donors, deceased donor LT, and small size liver graft [7,12-16].

Interacting with the genetic material of activated T-cell signaling in the β-cells, calcineurin inhibitors (CNIs) are thought to be diabetogenic through their action on the genetic material accountable for adequate β-cells function [17]. Furthermore, corticosteroids administered in high doses peri-operatively can inhibit β-cells function and alter the secretion of insulin, in addition to their apoptotic effect and their role in insulin-resistance induction. For instance, in the setting of kidney transplantation, Hecking et al. stipulated that the protection of β-cells from the pharmacologic stress peri-operatively may constitute an essential step in preventing insulin resistance and the emergence of NODAT [18]. Nam and his colleagues suggested that the most impactful factor in triggering NODAT is the termination of insulin secretion through damaging the β-cells via immunosuppressive therapies rather than inducing insulin resistance [19]. Hagen and her team demonstrated in their prospective cohort that impaired insulin secretion seems to be the major mechanism in the development of NODAT after renal transplantation and that normalization of glucose intolerance is associated with improved insulin secretion [20]. At the same time, Ekstrand et al. concluded in their early study that both insulin deficiency and resistance are directly involved in the pathogenesis of NODAT [21].

CNIs, namely TAC and cyclosporine A (CsA), are still considered the first-line agents for immunosuppression in LT recipients post-operatively over the past two decades [22]. Although TAC is well-known to provide a more desirable effect on the liver graft's long-term survival, recent studies are more and more revealing its direct impact on the emergence of NODALT and emphasizing its diabetogenicity compared to CsA, in addition to neurotoxicity and nephrotoxicity [23]. It has been reported that early minimization of CNIs after LT can be realized in the recipients without any serious long-term sequela and with low complications rates [24]. For instance, the current practice targets a TAC level within the range of 10-15 ng/mL during the first month after transplantation, then 5-10 ng/mL after that, while Jia and his colleagues suggested that levels as low as 5-7 ng/mL were sufficient to assure the safety and efficiency of the regimen [25,26]. However, there is a need for additional evidence to support this hypothesis.

On the other hand, several studies had shown that corticosteroids therapy might be considered an independent risk factor for the development of NODALT in liver transplant recipients [8,11,27-29]. Although used traditionally as an adjuvant immunosuppressive agent in LT recipients, some studies reveal significant evidence of the safety of their avoidance on the graft survival, thus the risen question about the risk-benefit ratio of their use during the PT period [30-32]. Therefore, this review aims to find the ultimate immunosuppressive therapy consisting of CNIs and corticosteroids after LT preventing the emergence of NODALT without compromising on the graft survival or functionality.

## Review

Methods

Search Strategy

We searched the PubMed database using the following MeSH keywords in multiple combinations: "Liver Transplantation," "Diabetes Mellitus," "NODM", "Tacrolimus," "Cyclosporine A," and "Steroids." In addition, we used the same keywords for regular searches in PubMed database and Google Scholar.

Inclusion/Exclusion Criteria

Types of patients and conditions: The etiologies that required LT in the first place were overlooked, and so were different medical and surgical histories, except that studies dealing with already diabetic patients were eliminated and only the ones reporting NODALT were taken in consideration. Relevant studies involving patients from all ages, sexes, races, and socioeconomic statuses were included.

Types of outcomes: We included studies that detected a noticeable effect of immunosuppressive regimens or their changing/discontinuing on the development/recovery of NODALT.

Types of studies: Only human studies relevant to the research target published in peer-reviewed journals between 2010 and 2020 were included. There were no geographical restrictions, but only English articles were included. Similar traditional review articles were eliminated in order to emphasize new significant findings as much as possible.

Study Records

Studies were screened based on their titles and abstracts and their eligibility criteria and relevance to the research goal. Full-text articles were reviewed in case of uncertainty. The chosen studies were then collected, stored and organized on Mendeley software, where duplicates were eventually eliminated.

Ethical Issue

All the selected studies were accessed ethically and legally on PubMed.

Discussion

The pathophysiology of NODALT and type 2 diabetes mellitus is very similar [33], but some transplant-related risk factors may trigger NODALT. For instance, multiple stress factors affecting pancreatic β-cells peri-operatively, such as the administration of high doses of corticosteroids, the initiation of CNIs therapy, the surgical and metabolic stress, and weight gain related to decreased physical activity, play an impactful role in inducing NODALT. Notably, the apoptosis of pancreatic β-cells and the progressive failure of pancreatic β-cells islets could explain the pathophysiology hiding behind the emergence of NODALT [34]. This section will discuss the best strategy of using CINs and corticosteroids to avoid NODALT without compromising graft survival.

CNIs: The Revolutionary Pills

**Figure 1:**
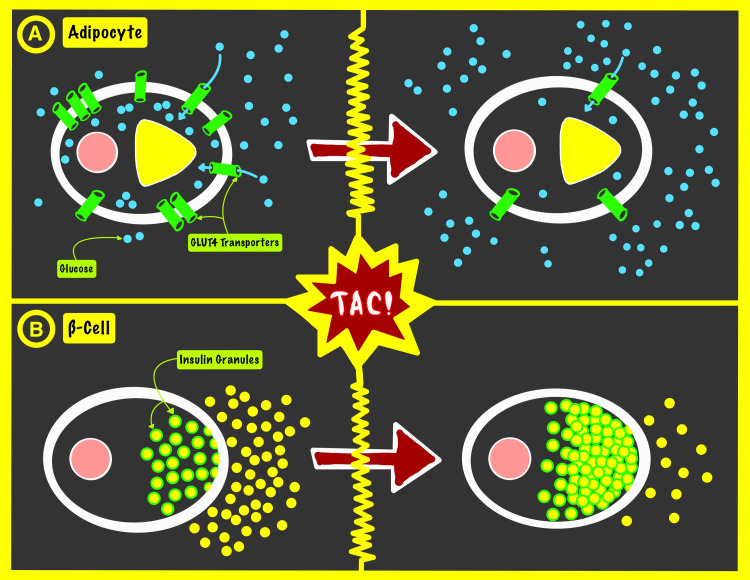
The effect of tacrolimus (TAC) on glycogen storage via regulating the expression of the glucose transporter 4 (GLUT4) proteins on adipocytes (A) and on the insulin degranulation from ß-cells (B). TAC: tacrolimus; ß-cell: pancreatic beta-cell; GLUT4: glucose transporter 4. This illustration was originally created by our team.

Since their introduction to the world of organ transplantation, CNIs have made a tremendous and major impact on the success of different types of solid organ transplantations and grafts' long-term maintenance. While recent studies questioned these drugs for a possible role in inducing NODALT, this hypothesis was relatively proven. TAC affects intracellular calcium metabolism, insulin granule degradation, and glucose transporters and has a more diabetogenic effect than CsA (Figure 1) [35,36].

A case-control study conducted by Terto et al. found that TAC, being the most common maintenance immunosuppressive agent used PT, was significantly associated with the emergence of NODALT during a mean time of five months PT [37]. Furthermore, Song and his colleagues went in-depth and assessed the blood concentration of tacrolimus (cTAC) systematically and established a significant relationship between the mean trough cTAC on the sixth-month PT and the onset of NODALT in two different studies with similar results. For instance, their 2018 study suggested that the mean cTAC at the sixth-month post-operatively was higher in recipients with NODALT (8.7 ng/mL ± 3.5) than that in the recipients without NODALT (4.7 ng/mL ± 3.5) and that a cut-off value of mean cTAC of 5.9 ng/mL was identified as an independent risk factor for NODALT [38]. Furthermore, the 2016 study suggested that the mean cTAC was higher in the NODALT group (7.66 ± 3.41 ng/mL) than in the non-NODALT group (4.47 ± 2.22 ng/mL), and a cut-off cTAC of 5.89 ng/mL was identified as predictive of NODALT, noting that they analyzed blood glucose data after six months to avoid the residual effects of corticosteroids on the recipients [39].

On the other hand, Liu et al. declare that the association between five commonly used immunosuppressant prescriptions and NODALT, including mycophenolate mofetil (MMF), showed that TAC has significantly higher correlations with NODALT than others. For instance, the incidence of NODALT was observed to be the highest in regimen CsA+TAC+MMF, followed respectively by regimen TAC+MMF and regimen CsA+MMF, noting that the development of NODALT appeared in most cases within one year after LT [40]. The overall recipients' survival was dependent significantly on the immunosuppressive regimen used (CsA+MMF > TAC+MMF > CsA+TAC+MMF). Abe and colleagues, in their retrospective cohort, highlighted that all of their patients with NODALT at the time of diagnosis were treated with TAC and that four of eight patients who developed NODALT recovered from it by switching from TAC to CsA [41]. Those four patients had transient DM that was reversible by withholding the stipulated culprit agent, i.e., TAC, whereas the other four patients with persistent DM required insulin therapy. On the same page, First et al. found that NODALT incidence was higher significantly with TAC than CsA. There was no difference between the two used formulations of TAC throughout the study (slow release and extended release) [42].

Despite the incidence of adverse effects such as chronic renal failure, hypertension, de novo malignancy, and NODM, TAC remains the cornerstone of preventing rejection after LT, and these adverse effects have led to a focus shift from acute cellular rejection and short-term post-transplant survival to long-term management of complications. Furthermore, strategies to minimize CNI exposure have been developed, and despite that TAC has a narrow therapeutic index and that its oral bioavailability is highly variable between individuals, the conversion from the TAC twice daily (BID) to TAC once daily (OD) formulation in stable LT patients is deemed safe, and it will increase the compliance and the patients' quality of life [43]. It is noteworthy to emphasize the serious possibility of developing diabetic ketoacidosis (DKA) in recipients on TAC maintenance therapy as shown by Masood et al., thus reducing the TAC dose and maintaining a cTAC as low as possible in the PT setting may contribute to the prevention of this life-threatening metabolic complication that the drug itself may trigger [44].

Lorho et al. studied the conversion from TAC to CsA in the case of established NODALT. They deduced that the recipients suffering from NODALT might benefit from this conversion through an improvement in the level of glucose metabolism. The target dose of CsA serum concentration (cCsA) was 500-700 ng/mL [45]. Table 1 shows the summary of the studies discussing the effect of the TAC-based immunosuppressive regimen.

**Table 1:** Summary of studies discussing the effect of tacrolimus-based immunosuppressive regimen on the development of new-onset diabetes mellitus (NODM). NODALT: new-onset diabetes mellitus after liver transplantation; TAC: tacrolimus, MMF: mycophenolate mofetil;  AZA: azathioprine; LT: liver transplantation; cTAC: serum concentration of tacrolimus; CsA: cyclosporine A; LFTs: liver function tests; CNI: calcineurin inhibitor; DKA: diabetic ketoacidosis; ADA: American Diabetes Association; BID: twice daily; OD: once daily.

Author (year)	Type	Aim	Immunosuppressive Protocol (excluding steroids)	Conclusion
Terto et al. (2019) [37]	Case-Control	To assess the risk factors associated with NODALT.	﻿TAC: 100% of NODALT patients and 98.3% of the control group. MMF: 75.9% of TAC group vs. 60.7% of the control group. AZA: 0.9% of the control group.	﻿Pre-existing systemic arterial hypertension and the associated use of MMF and TAC increased the risk of NODALT.
Song et al. (2018) [38]	Retrospective Cohort	﻿To investigate the association between TAC blood concentration and the risk of NODALT development after living donor LT.	TAC: ﻿the initial dose was 0.05-0.10 mg/kg per day and tapered according to LFTs and cTAC. MMF: ﻿1.0 g/day and 1.5 g/day initially discontinued when severe side effects occurred and long-term survivors with stable graft function after six mo after LT. Rapamycin: ﻿as an alternative to MMF or an auxiliary for liver tumor at a dose of 1 mg/day.	﻿Higher mean cTAC at the sixth month post-operatively is related to increased risk of NODALT in LT recipients.
Song et al. (2016) [39]	Retrospective Cohort	﻿To investigate the impact of minimum TAC on NODALT.	TAC: ﻿the initial dose was 0.05-0.10 mg/kg per day and tapered according to LFTs and cTAC. MMF: ﻿1.0 g/day and 1.5 g/day initially discontinued when severe side effects occurred and long-term survivors with stable graft function after six mo after LT. Rapamycin: ﻿as an alternative to MMF or an auxiliary for liver tumor at a dose of 1 mg/day.	﻿A minimal TAC regimen can decrease the risk of long-term NODALT. Maintaining a cTAC value below 5.89 ng/mL after LT is safe and beneficial.
Liu et al. (2017) [40]	Retrospective Cohort	﻿To clarify the effects of immunosuppressive regimens on NODALT.	﻿TAC+MMF: 64.5%, CsA+MMF: 7.6%, CsA+TAC+MMF: 3.8%.	﻿TAC-based immunosuppression contributes to higher NODALT incidence than CsA-based regimen, and TAC-CsA conversion due to any causes might lead to worse clinical outcomes.
Abe et al. (2014) [41]	Retrospective Cohort	﻿To elucidate the risk factors for the development of NODALT and those for progressive glucose intolerance in adult living-donor liver transplant recipients.	All of the NODALT patients were being treated with TAC at the time of diagnosis.	Switching the CNI from TAC to CsA allowed one-half of the patients (4/8) to withdraw from insulin-dependent therapy.
First et al. (2013) [42]	Prospective Cohort	﻿To propose a new approach to defining NODALT based on the ADA criteria.	TAC/CsA	44% to 45% in liver transplant recipients treated with TAC. NODALT incidence was significantly higher with TAC than CsA; there was no difference between the two TAC formulations.
Beckebaum et al. (2011) [43]	Prospective Cohort	To determine the efficacy, safety, and immunosuppressant adherence in 125 stable LT patients converted from TAC BID to TAC OD	TAC	Conversion to TAC OD is safe, enhances immunosuppressant adherence and should be accompanied by a close TAC level monitoring during the initial period.
Masood et al. (2011) [44]	Case Report	﻿ To describe two cases in solid-organ transplant recipients (Liver/kidney) developing DKA in patients receiving TAC in the post-transplant setting.	TAC / CsA	﻿Clinicians should be cognizant of the possibility of hyperglycemic crisis presenting as sudden onset of DKA in patients receiving TAC.
Lorho et al. (2011) [45]	Prospective Pilot	﻿To evaluate the impact of conversion from TAC to CsA in liver transplant patients presenting NODALT.	CsA: 5 mg/kg ﻿for first three days then 700-900 ng/mL in the four-six months following transplantation or 500-700 ng/mL thereafter.	﻿Liver transplant patients with NODALT may benefit from conversion to CsA from TAC through improved glucose metabolism.

Corticosteroids: The Unnecessary Culprit?

Corticosteroids are considered an essential adjuvant in contemporary practice for maintaining immunosuppression after LT, despite their very diverse and rich profile of adverse effects and serious bluster to the graft and the recipient as a whole. Weiler et al. listed the steroid withdrawal protocols used nowadays after transplantation, which are: long-term steroid withdrawal protocol (six-12 months), early steroid withdrawal protocol (ESWP) (within three months), very early steroid withdrawal protocol (two weeks), steroid almost-avoidance protocol (elimination within one week), and steroid complete-avoidance protocol. To date, there is no data reporting the application of a steroid almost-avoidance protocol or a steroid complete-avoidance protocol as they are associated with a high risk of acute graft rejection and loss. However, Weiler and his colleagues applied the very early steroid withdrawal protocol in their cohort, and the results are found not to be very encouraging. For instance, NODALT rates were comparable between the steroids group and the placebo one. In contrast, chronic rejection rates were significantly higher in addition to reduced safety in the long run [46].

Castedal et al. demonstrated that applying a steroid-free low-dose TAC-based immunosuppression in the PT period decreased the incidence of NODALT significantly and proved to be safe [47]. The steroids protocol was tapered over 180 days (ESWP), but maintenance doses were negligible. Roughly the same conclusion was reported by Ju et al., where they conducted a cohort quite similar to Castedal's and his team with a similar ESWP but prospectively [48]. Jiu and his team concluded that a 24-hour steroid avoidance is safe and efficient when added in adjunction to TAC maintenance without any significant risk of acute graft rejection. Maybe the most interesting study conducted on this issue was the prospective cohort of Kim and his team in 2012 [49]. It is the only study that applied a very early steroid withdrawal protocol in the past 10 years, and it showed impressive results. For instance, the study showed a safe profile of ditching steroids as early as 14 days PT in old age recipients and a significant decrease in NODALT rates. Table 2 gives the summary of the studies discussing the effect of different corticosteroid immunosuppressive regimens.

**Table 2:** Summary of studies discussing the effect of different corticosteroid immunosuppressive regimens on the development of new-onset diabetes mellitus (NODM). NODALT: new-onset diabetes mellitus after liver transplantation; IS: immunosuppression; TAC: tacrolimus; D: post-operative day; LDLT: living donor liver transplantation; NODM: new-onset diabetes mellitus; PO: per os; IV: intravenously; OD: once daily.

Author (year)	Type	Aim	Steroids Protocol	Relevant Conclusion
Castedal et al. (2018) [47]	Retrospective Cohort	To compare the incidence of transient as well as persistent NODALT, rejection rate, and patient and graft survival between patients receiving steroid-based and steroid-free maintenance IS.	Methylprednisolone: D0: 500-1000 mg once intraoperatively (IV). Prednisolone: D1-D4: tapering from 200 mg to 10 mg BID (IV), D5-D30: tapering to 5-10 mg OD (PO), D31-D90: 5 mg OD (PO).	﻿Steroid-free low-dose TAC-based immunosuppression following LT is safe and decreases the incidence of NODALT significantly.
Ju et al. (2012) [48]	Prospective Cohort	﻿To investigate the efficacy and safety of an immunosuppressive regimen of steroid avoidance in combination with induction therapy and TAC in liver transplant recipients.	Methylprednisolone: D0: 500 mg once intraoperatively (IV), D1: 240 mg once (IV), D2-D7: 10 mg OD (PO), D8-D9: 48 mg OD (PO), reduced by 8 mg every three days and maintained at four mg/d after that, until it was completely stopped three months PT.	﻿Twenty-four-hour steroid avoidance combined with induction therapy and tacrolimus maintenance is a safe and efficient immunosuppression strategy that can significantly reduce post-transplant infections and other complications owing to long-term use of steroids without increasing the risk of acute rejection.
Kim et al. (2012) [49]	Prospective Cohort	﻿﻿Identify a patient subgroup who would benefit concerning NODALT by an early steroid withdrawal regimen after LDLT.	Methylprednisolone: D0: 500 mg once (IV), tapered to 20 mg PO on D6, then off by D14.	﻿ESWR can safely reduce the incidence of NODM after LDLT in old-age recipients.
Weiler et al. (2010) [46]	Prospective Cohort	﻿To evaluate early steroid-free immunosuppression in liver transplant patients.	Methylprednisolone: D0: 1000 mg once intraoperatively (IV), D1-D14: tapering from 100 mg to 12 mg OD (IV), D15-D60: 12 mg OD (PO), D61-D180: 8 mg OD (PO).	Early tapering down of steroids to a TAC monotherapy is possible with comparable acute rejection rates During steroid therapy, NODALT and hypercholesterolemia are cumulative. These side effects are reversible.

The Ultimate Immunosuppressive Strategy

**Figure 2:**
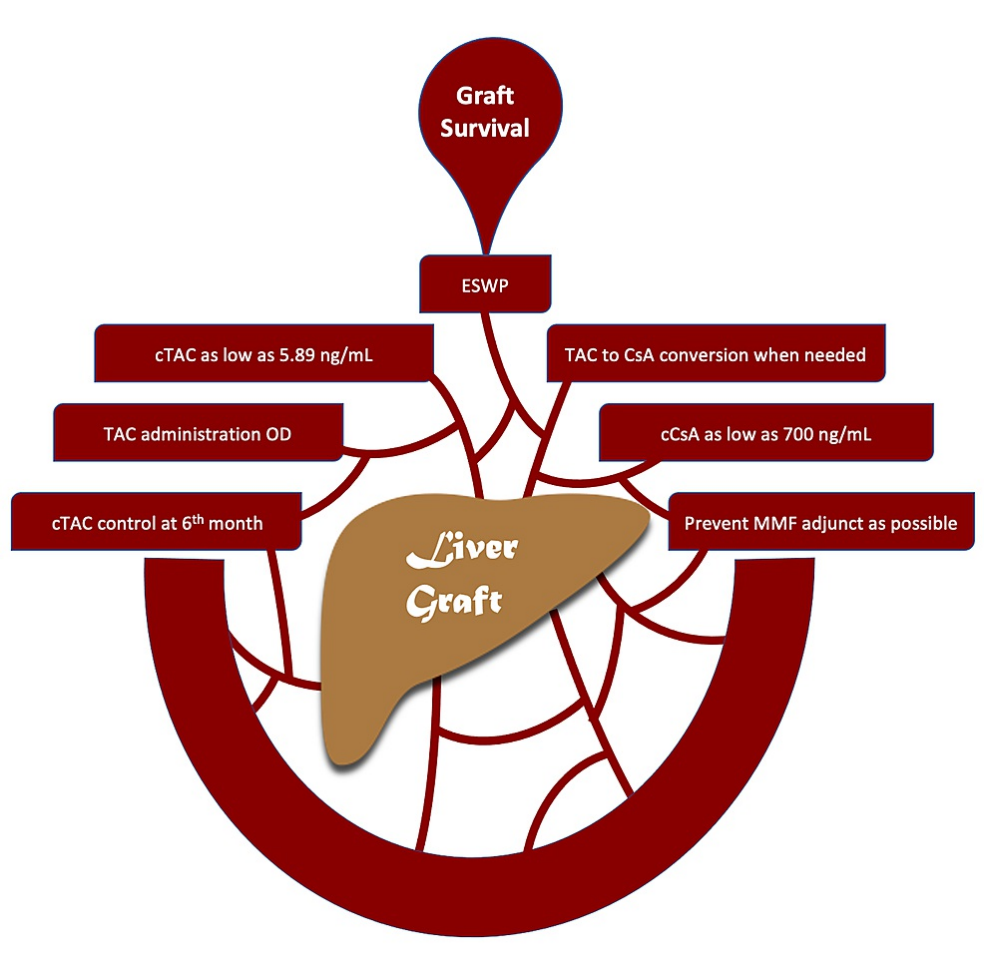
Summary of the ultimate immunosuppressive strategy. ESWP: early steroid withdrawal protocol; TAC: tacrolimus; cTAC: serum concentration of tacrolimus; CsA: cyclosporine A; cCsA: serum concentration of cyclosporine A; MMF: mycophenolate mofetil; OD: once daily. This illustration was originally created by our team.

Many steps that can be considered when pharmacologically managing LT recipients post-operatively and leading to the least exposure to diabetogenic agents are highly encouraged according to the current evidence (Figure 2).

It is still controversial to eliminate corticosteroids from the immunosuppressive regimen of transplant recipients in general. However, the successful application of ESWP can constitute the starting point for a steroid-free future of organ transplantation, especially LT, although the current evidence is limiting the positive results of this protocol to the old population of recipients. As a wrap-up, it is considered safe to start the LT recipient on TAC post-operatively and maintain a minimal cTAC as low as 5.89 ng/mL in addition to low doses of corticosteroids according to an ESWP within two weeks of the surgery. Administering TAC OD instead of BID carries an additional benefit of medication adherence and may improve the patients' quality of life. What's more important is monitoring cTAC throughout the PT period since it is critical to the metabolic prognosis. An elevated mean value at the sixth month PT could indicate a relative risk of developing NODALT. TAC to CsA conversion is a safe secret weapon that can be used to detect high blood glucose readings, and the blood concentration of CsA should range around 700 ng/mL.

Strengths and Limitations

This review constitutes a concise summary of the existing literature concerning the matter at hand, highlighting the latest tips and techniques used by experts in the field of LT to prevent/treat NODALT while trying as much as possible to maintain the integrity of the liver graft. In contrast, its major weakness resides in generalizing the population included and not taking into consideration the different etiologies of liver failure in the first place (viral infection, cancer, cirrhosis, autoimmune process, ischemic injury, etc.), which may affect widely the mechanism of developing of NODM, and also the response to different immunosuppressive agents. This review was also limited in its time frame (2010-2020), which may have led to missing some important studies related to the research question, in addition to eliminating animal studies, which may include some significant scientific input for future trials and strategies.

## Conclusions

The ultimate non-diabetogenic immunosuppressive therapy in LT recipients should always be second to the graft's safety and longevity by preventing failure due to an immune reaction. At the same time, NODALT is one of the metabolic factors that could jeopardize the mission's success. Current evidence resonates with the ability to lower the risks of developing NODALT by lowering the exposure to TAC and corticosteroids after successful transplantation. Further studies are requested to assess the safety of ESWP on a larger scale, in addition to the safety of low cTAC in the long term, years after transplantation, added to the levels of CsA when the conversion is needed and indicated.

## References

[1] (2020). United network for organ sharing 2009 annual report. https://unos.org/wp-content/uploads/unos/AnnualReport2009.pdf.

[2] Meirelles Júnior RF, Salvalaggio P, Rezende MB (2015). Liver transplantation: history, outcomes and perspectives. Einstein (Sao Paulo).

[3] Anderson AL, Lewis DA, Steinke DT, Ranjan D, Smith KM, Clifford TM (2009). Effects of hyperglycemia on the development of new-onset diabetes after liver transplantation. Prog Transplant.

[4] Sarno G, Mehta RJ, Guardado-Mendoza R, Jimenez-Ceja LM, De Rosa P, Muscogiuri G (2013). New-onset diabetes mellitus: predictive factors and impact on the outcome of patients undergoing liver transplantation. Curr Diabetes Rev.

[5] Moon JI, Barbeito R, Faradji RN, Gaynor JJ, Tzakis AG (2006). Negative impact of new-onset diabetes mellitus on patient and graft survival after liver transplantation: long-term follow up. Transplantation.

[6] American Diabetes Association (2019). 2. Classification and diagnosis of diabetes: standards of medical care in diabetes-2019. Diabetes Care.

[7] Yadav AD, Chang YH, Aqel BA (2013). New-onset diabetes mellitus in living donor versus deceased donor liver transplant recipients: analysis of the UNOS/OPTN database. J Transplant.

[8] Saliba F, Lakehal M, Pageaux GP (2007). Risk factors for new-onset diabetes mellitus following liver transplantation and impact of hepatitis C infection : an observational multicenter study. Liver Transpl.

[9] Saab S, Shpaner A, Zhao Y (2006). Prevalence and risk factors for diabetes mellitus in moderate term survivors of liver transplantation. Am J Transplant.

[10] Oufroukhi L, Kamar N, Muscari F (2008). Predictive factors for posttransplant diabetes mellitus within one-year of liver transplantation. Transplantation.

[11] Kuo HT, Sampaio MS, Ye X, Reddy P, Martin P, Bunnapradist S (2010). Risk factors for new-onset diabetes mellitus in adult liver transplant recipients, an analysis of the Organ Procurement and Transplant Network/United Network for Organ Sharing database. Transplantation.

[12] Parvizi Z, Azarpira N, Kohan L, Darai M, Kazemi K, Parvizi MM (2014). Association between E23K variant in KCNJ11 gene and new-onset diabetes after liver transplantation. Mol Biol Rep.

[13] Marchetti P (2005). New-onset diabetes after liver transplantation: from pathogenesis to management. Liver Transpl.

[14] Varghese J, Reddy MS, Venugopal K (2014). Tacrolimus-related adverse effects in liver transplant recipients: its association with trough concentrations. Indian J Gastroenterol.

[15] Pham PT, Pham PM, Pham SV, Pham PA, Pham PC (2011). New onset diabetes after transplantation (NODAT): an overview. Diabetes Metab Syndr Obes.

[16] Ling Q, Xie H, Lu D (2013). Association between donor and recipient TCF7L2 gene polymorphisms and the risk of new-onset diabetes mellitus after liver transplantation in a Han Chinese population. J Hepatol.

[17] Heit JJ, Apelqvist AA, Gu X, Winslow MM, Neilson JR, Crabtree GR, Kim SK (2006). Calcineurin/NFAT signalling regulates pancreatic beta-cell growth and function. Nature.

[18] Hecking M, Werzowa J, Haidinger M (2013). Novel views on new-onset diabetes after transplantation: development, prevention and treatment. Nephrol Dial Transplant.

[19] Nam JH, Mun JI, Kim SI (2001). Beta-cell dysfunction rather than insulin resistance is the main contributing factor for the development of postrenal transplantation diabetes mellitus. Transplantation.

[20] Hagen M, Hjelmesaeth J, Jenssen T, Morkrid L, Hartmann A (2003). A 6-year prospective study on new onset diabetes mellitus, insulin release and insulin sensitivity in renal transplant recipients. Nephrol Dial Transplant.

[21] Ekstrand AV, Eriksson JG, Grönhagen-Riska C, Ahonen PJ, Groop LC (1992). Insulin resistance and insulin deficiency in the pathogenesis of posttransplantation diabetes in man. Transplantation.

[22] U.S. Multicenter FK506 Liver Study Group (1994). A comparison of tacrolimus (FK 506) and cyclosporine for immunosuppression in liver transplantation. N Engl J Med.

[23] Wiesner RH, Fung JJ (2011). Present state of immunosuppressive therapy in liver transplant recipients. Liver Transpl.

[24] Kong Y, Wang D, Shang Y, Liang W, Ling X, Guo Z, He X (2011). Calcineurin-inhibitor minimization in liver transplant patients with calcineurin-inhibitor-related renal dysfunction: a meta-analysis. PLoS One.

[25] Boillot O, Seket B, Dumortier J, Pittau G, Boucaud C, Bouffard Y, Scoazec JY (2009). Thymoglobulin induction in liver transplant recipients with a tacrolimus, mycophenolate mofetil, and steroid immunosuppressive regimen: a five-year randomized prospective study. Liver Transpl.

[26] Jia JJ, Lin BY, He JJ (2014). ''Minimizing tacrolimus'' strategy and long-term survival after liver transplantation. World J Gastroenterol.

[27] Friedman EA, Shyh TP, Beyer MM, Manis T, Butt KM (1985). Posttransplant diabetes in kidney transplant recipients. Am J Nephrol.

[28] Arner P, Gunnarsson R, Blomdahl S, Groth CG (1983). Some characteristics of steroid diabetes: a study in renal-transplant recipients receiving high-dose corticosteroid therapy. Diabetes Care.

[29] David DS, Cheigh JS, Braun Jr DW, Fotino M, Stenzel KH, Rubin AL (1980). HLA-A28 and steroid-induced diabetes in renal transplant patients. JAMA.

[30] Lerut JP (2003). Avoiding steroids in solid organ transplantation. Transpl Int.

[31] Segev DL, Sozio SM, Shin EJ (2008). Steroid avoidance in liver transplantation: meta-analysis and meta-regression of randomized trials. Liver Transpl.

[32] Eason JD, Nair S, Cohen AJ, Blazek JL, Loss GE Jr (2003). Steroid-free liver transplantation using rabbit antithymocyte globulin and early tacrolimus monotherapy. Transplantation.

[33] Davidson J, Wilkinson A, Dantal J (2003). New-onset diabetes after transplantation: 2003 international consensus guidelines. Proceedings of an international expert panel meeting. Barcelona, Spain, 19 February 2003. Transplantation.

[34] Chakkera HA, Knowler WC, Devarapalli Y (2010). Relationship between inpatient hyperglycemia and insulin treatment after kidney transplantation and future new onset diabetes mellitus. Clin J Am Soc Nephrol.

[35] Pereira MJ, Palming J, Rizell M, Aureliano M, Carvalho E, Svensson MK, Eriksson JW (2014). Cyclosporine A and tacrolimus reduce the amount of GLUT4 at the cell surface in human adipocytes: increased endocytosis as a potential mechanism for the diabetogenic effects of immunosuppressive agents. J Clin Endocrinol Metab.

[36] Øzbay LA, Smidt K, Mortensen DM, Carstens J, Jørgensen KA, Rungby J (2011). Cyclosporin and tacrolimus impair insulin secretion and transcriptional regulation in INS-1E beta-cells. Br J Pharmacol.

[37] Terto SV, Araújo ST, Negreiros FD (2019). Risk Factors Associated With New-Onset Diabetes After Liver Transplant: A Case Control Study. Transplant Proc.

[38] Song JL, Li M, Yan LN, Yang JY, Yang J, Jiang L (2018). Higher tacrolimus blood concentration is related to increased risk of post-transplantation diabetes mellitus after living donor liver transplantation. Int J Surg.

[39] Song JL, Gao W, Zhong Y (2016). Minimizing tacrolimus decreases the risk of new-onset diabetes mellitus after liver transplantation. World J Gastroenterol.

[40] Liu FC, Lin HT, Lin JR, Yu HP (2017). Impact of immunosuppressant therapy on new-onset diabetes in liver transplant recipients. Ther Clin Risk Manag.

[41] Abe T, Onoe T, Tahara H (2014). Risk factors for development of new-onset diabetes mellitus and progressive impairment of glucose metabolism after living-donor liver transplantation. Transplant Proc.

[42] First MR, Dhadda S, Croy R, Holman J, Fitzsimmons WE (2013). New-onset diabetes after transplantation (NODAT): an evaluation of definitions in clinical trials. Transplantation.

[43] Beckebaum S, Iacob S, Sweid D (2011). Efficacy, safety, and immunosuppressant adherence in stable liver transplant patients converted from a twice-daily tacrolimus-based regimen to once-daily tacrolimus extended-release formulation. Transpl Int.

[44] Masood MQ, Rabbani M, Jafri W, Habib M, Saleem T (2011). Diabetic ketoacidosis associated with tacrolimus in solid organ transplant recipients. J Pak Med Assoc.

[45] Lorho R, Hardwigsen J, Dumortier J (2011). Regression of new-onset diabetes mellitus after conversion from tacrolimus to cyclosporine in liver transplant patients: results of a pilot study. Clin Res Hepatol Gastroenterol.

[46] Weiler N, Thrun I, Hoppe-Lotichius M, Zimmermann T, Kraemer I, Otto G (2010). Early steroid-free immunosuppression with FK506 after liver transplantation: long-term results of a prospectively randomized double-blinded trial. Transplantation.

[47] Castedal M, Skoglund C, Axelson C, Bennet W (2018). Steroid-free immunosuppression with low-dose tacrolimus is safe and significantly reduces the incidence of new-onset diabetes mellitus following liver transplantation. Scand J Gastroenterol.

[48] Ju WQ, Guo ZY, Ling X (2012). Twenty-four hour steroid avoidance immunosuppressive regimen in liver transplant recipients. Exp Clin Transplant.

[49] Kim YK, Lee KW, Kim SH, Cho SY, Han SS, Park SJ (2012). Early steroid withdrawal regimen prevents new-onset diabetes mellitus in old-age recipients after living donor liver transplantation. World J Surg.

